# Updates on SPOP Gene Mutations in Prostate Cancer and Computational Insights From TCGA cBioPortal Database

**DOI:** 10.1155/sci5/4084224

**Published:** 2025-05-20

**Authors:** Suleiman Zakari, Solomon O. Rotimi, Chandra Tatsha Bholah, Olubanke O. Ogunlana

**Affiliations:** ^1^Department of Biochemistry, College of Science and Technology, Covenant University, Ota, Ogun, Nigeria; ^2^Cancer Genomics Laboratory, Covenant Applied Informatics and Communication-Africa Centre of Excellence (CApIC-ACE), Ota, Ogun, Nigeria; ^3^Department of Biochemistry, College of Medicine, Federal University of Health Sciences, Otukpo, Benue, Nigeria; ^4^School of Health Sciences, University of Technology Mauritius, La Tour Koenig, Pointe-Aux-Sables, Port Louis, Mauritius

**Keywords:** androgen receptor, prostate cancer, SPOP gene, SPOP mutations, TCGA cBioPortal, therapeutic targets

## Abstract

Speckle-type pox virus and zinc finger protein (SPOP) has emerged as a key focus in prostate cancer research due to its critical role in regulating the androgen receptor (AR) signaling pathway. This review aims to comprehensively summarize current knowledge on SPOP gene mutations in prostate cancer, emphasizing their importance in disease characterization and identification of therapeutic targets. A systematic literature search was conducted across multiple databases, including PubMed, Web of Science, Scopus, and Google Scholar. In addition, this study uses computational approaches and data from the TCGA cBioPortal database to explore the landscape of SPOP mutations in prostate cancer. After screening 682 articles and following systematic selection steps, 56 high-quality articles were included. Computational analysis of TCGA cBioPortal data revealed a SPOP mutation prevalence of 5%-6%, along with significant alterations in AR signaling and epigenetic regulation. SPOP mutations disrupt substrate recognition, leading to dysregulation of downstream pathways such as AR signaling and chromatin remodeling. Notably, SPOP-mutant prostate cancers are mutually exclusive with TMPRSS2-ERG fusions and enriched for Wnt pathway alterations. Patients with SPOP mutations demonstrate prolonged responses to androgen deprivation therapy (ADT), although concurrent mutations in TP53 or DNA repair genes negatively impact outcomes. While their prognostic significance continues to evolve, their impact on the AR pathway highlights their potential as therapeutic targets. The clinical implications of SPOP mutations are substantial, as they are linked to variations in treatment response and disease progression, thus serving as valuable biomarkers for risk stratification and prognosis.

## 1. Introduction

Prostate cancer, a prevalent malignancy among men, continues to be a formidable global health challenge, demanding rigorous scientific inquiry to unravel its complexities and develop more effective therapeutic strategies [1]. The landscape of prostate cancer biology centers around the androgen receptor (AR) signaling pathway, which plays a pivotal role in disease development, progression, and therapeutic responses [2]. The spotlight in prostate cancer research has shifted to a protein with a seemingly innocuous acronym, Speckle-type POZ protein (SPOP), due to its profound influence on AR pathway regulation [3]. The discovery of recurrent SPOP mutations in prostate cancer has sparked a transformative paradigm shift in our understanding of this disease [4, 5]. In this review, we explore the implications of SPOP mutations in prostate cancer, highlighting their significance for disease characterization and their potential as therapeutic targets. Prostate cancer represents a wide spectrum of clinical outcomes, ranging from indolent localized tumors that may not require immediate treatment to highly aggressive forms that rapidly metastasize and evade current therapeutic interventions. The heterogeneity in clinical behavior and response to treatment poses a substantial clinical challenge, prompting an urgent need for molecular insights that can inform more precise clinical management [6].

The AR signaling pathway is integral to normal prostate development and function, and its aberrant activation drives the initiation and progression of prostate cancer. The AR is a ligand-dependent transcription factor that regulates the expression of genes crucial for prostate cell growth and survival. AR signaling has been a focal point for therapeutic interventions in prostate cancer, leading to the development of androgen deprivation therapy (ADT) as a cornerstone treatment for advanced disease [7]. However, despite initial responses to ADT, most prostate cancers eventually progress to a more aggressive, castration-resistant state, where AR signaling remains a driving force behind tumor growth [8]. This transition highlights the dynamic adaptability of prostate cancer cells in the face of therapeutic pressure [9]. Consequently, the search for novel therapeutic targets and strategies to combat castration-resistant prostate cancer (CRPC) is of paramount importance.

SPOP, an E3 ubiquitin ligase substrate adaptor protein, plays a crucial role in maintaining cellular homeostasis by targeting specific substrates for ubiquitination and subsequent proteasomal degradation [10]. Among its substrates, the AR protein stands out as a key player in prostate cancer pathogenesis. The interaction between SPOP and AR occurs primarily through the substrate-binding Mec-17, Apx, and TRIP (MATH) domain of SPOP, which recognizes and facilitates the ubiquitination of AR. The discovery of SPOP mutations, particularly within the MATH domain, has unveiled a new layer of complexity in prostate cancer biology. These mutations compromise SPOP's ability to interact with and ubiquitinate AR, resulting in the stabilization and accumulation of the AR protein. The prevalence and functional consequences of SPOP mutations in prostate cancer are crucial for a comprehensive characterization of the disease.

The molecular consequences of SPOP mutations extend beyond AR dysregulation, encompassing alterations in various signaling pathways and cellular processes [11]. These effects have significant implications for disease progression, as SPOP-mutant prostate cancers often exhibit distinct clinical and molecular features. Understanding these alterations is essential for stratifying patients and tailoring treatment approaches to individualized needs. The clinical implications of SPOP mutations in prostate cancer are multifaceted. These mutations have been associated with both favorable and adverse clinical outcomes, depending on the specific context and co-occurring genetic alterations [12]. Elucidating the prognostic significance of SPOP mutations is essential for risk stratification and treatment decision-making. Furthermore, the unique vulnerabilities conferred by SPOP mutations have opened new avenues for targeted therapy development. Exploiting the dependence of SPOP-mutant prostate cancer cells on dysregulated AR signaling represents a promising therapeutic strategy.

In this comprehensive review, we delve into the multifaceted role of SPOP mutations in prostate cancer, exploring their prevalence, functional consequences, clinical implications, and potential as therapeutic targets. By unraveling the complex interplay between SPOP mutations and the AR signaling pathway, we aim to contribute to the ongoing efforts to improve our understanding of prostate cancer biology and enhance patient outcomes through more precise and effective therapeutic interventions.

## 2. Methods

A systematic literature review was conducted to identify relevant studies related to SPOP mutations in prostate cancer. The search was performed in multiple electronic databases, including PubMed, Scopus, Web of Science, and Google Scholar. The following search terms and Boolean operators were used: (“SPOP mutations” OR “SPOP gene alterations”) AND (“prostate cancer” OR “prostate carcinoma” OR “prostatic neoplasms”) AND (“androgen receptor” OR “AR signaling pathway” OR “androgen receptor regulation”).

### 2.1. Eligibility Criteria

#### 2.1.1. Inclusion Criteria

a. Relevance to SPOP mutations: Studies and articles must directly investigate or discuss SPOP mutations in the context of prostate cancer. This includes research focused on the molecular characterization of SPOP mutations and their implications in prostate cancer.
b. Publication date: Articles published from inception up to December 2024 were considered to ensure the inclusion of the most recent research.
c. Study type: Original research articles, reviews, meta-analyses, and clinical trials were included to provide a comprehensive overview of the topic.
d. Language: Articles published in English were included for review.
e. Data availability: Studies that provide sufficient data related to SPOP mutations, their prevalence, molecular mechanisms, disease characterization, or therapeutic implications were considered.

#### 2.1.2. Exclusion Criteria

a. Irrelevance: Studies not pertaining to SPOP mutations in prostate cancer or lack relevance to the research topic will be excluded.
b. Insufficient data: Articles that lack adequate data or details on SPOP mutations, their role in prostate cancer, or their impact on disease characterization and therapy will not be included.
c. Publication type: Editorials, commentaries, letters, conference abstracts, and non-peer-reviewed articles will be excluded.
d. Language barrier: Articles published in languages other than English will not be considered due to potential language barriers.
e. Outdated publications: Studies published before the inception of the electronic databases used for the search will be excluded to focus on recent research.

### 2.2. Screening and Selection

A two-stage study selection process was employed. Initially, titles and abstracts of retrieved articles were screened against the predefined eligibility criteria. Subsequently, full-text articles of potentially eligible studies were assessed for final inclusion. The initial search yielded a total of 682 articles across the selected databases. The screening and selection process was conducted in multiple stages to identify relevant studies for inclusion in the systematic review:

#### 2.2.1. Title and Abstract Screening

The titles of all retrieved articles were screened to exclude irrelevant studies. Duplicate articles were also removed at this stage. After title screening, 398 articles remained. The abstracts of the remaining articles were reviewed to assess their relevance to the research topic. Articles that did not meet the inclusion criteria, such as those unrelated to SPOP mutations in prostate cancer or lacking sufficient data, were excluded. After abstract review, 147 articles remained.

#### 2.2.2. Full-Text Evaluation

The full texts of the 147 selected articles were thoroughly evaluated. Articles that did not provide information on SPOP mutations in prostate cancer, did not contribute to disease characterization, or did not address therapeutic implications were excluded. A total of 87 articles met the inclusion criteria.

#### 2.2.3. Quality Assessment

The selected articles underwent a quality assessment to evaluate the reliability and validity of the data presented. Studies with methodological flaws or inadequate reporting were excluded. A total of 56 articles were deemed sufficient for inclusion in the systematic review.

#### 2.2.4. Final Literature Selection

After rigorous screening and selection, 56 articles were included in the systematic review. These articles were chosen based on their relevance to SPOP mutations in prostate cancer, their contributions to disease characterization, and their insights into therapeutic implications related to the AR signaling pathway. These selected articles form the basis for the comprehensive analysis presented in this review. The systematic review process adhered to established guidelines for systematic reviews and was designed to ensure the inclusion of high-quality, pertinent literature while excluding irrelevant or low-quality studies.

## 3. Results

**Figure 1:**
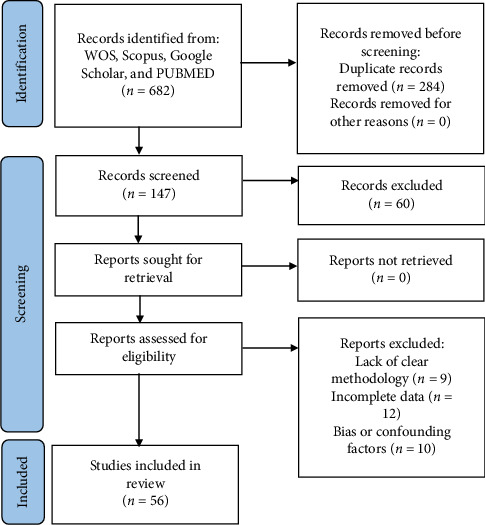
Study flowchart showing search output.

A systematic literature review was conducted to investigate the role of SPOP mutations in prostate cancer (Figure 1). The search encompassed major electronic databases, including PubMed, Scopus, Web of Science, and Google Scholar. The search terms employed included “SPOP mutations” and “SPOP gene alterations” in conjunction with “prostate cancer,” “prostate carcinoma,” or “prostatic neoplasms.” Additionally, terms related to the AR signaling pathway, such as “Androgen Receptor” and “androgen receptor regulation,” were incorporated using Boolean operators. The initial search identified a comprehensive pool of 682 articles across the selected databases, constituting the starting point for the systematic review. To streamline the selection process, the titles of all retrieved articles underwent rigorous screening. Irrelevant studies were excluded during this phase, and duplicate articles were eliminated. Following title screening, 398 articles remained for further evaluation. A more in-depth assessment was conducted by reviewing the abstracts of the remaining articles. Articles that did not align with the predefined inclusion criteria, such as those lacking relevance to SPOP mutations in prostate cancer or those with insufficient data, were excluded. This stage yielded 147 articles for continued consideration. The 147 selected articles underwent a comprehensive evaluation of their full texts. Articles that did not provide pertinent information on SPOP mutations in prostate cancer, did not contribute to disease characterization, or did not address therapeutic implications were excluded. This rigorous evaluation process resulted in identifying a subset of 87 articles that met the predefined inclusion criteria. The selected articles underwent a quality assessment to ensure the data's reliability and validity. Studies with methodological flaws or inadequate reporting were excluded to maintain the overall quality of the review. Ultimately, a total of 56 articles were considered of sufficient quality for inclusion in the systematic review. After meticulous screening, selection, and quality assessment, a final set of 56 articles were included in the systematic review. These articles were chosen based on their direct relevance to SPOP mutations in prostate cancer, their contributions to disease characterization, and their insights into therapeutic implications related to the AR signaling pathway.

## 4. Discussion

### 4.1. SPOP Gene and Protein

**Figure 2:**
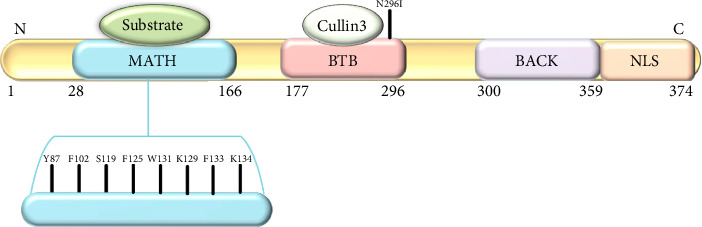
Structure of SPOP protein.

The SPOP gene is a gene in humans that encodes the SPOP protein. The SPOP gene is located on chromosome 17q21.2 [5]. It plays a crucial role in cellular regulation, particularly in protein degradation and post-translational modifications. Understanding the SPOP gene and its protein product is essential for grasping its significance in various cellular processes and its involvement in diseases, especially cancer. The SPOP gene is situated on chromosome 17, specifically at the long arm of the chromosome, known as 17q21.2. This genomic location is important because alterations or mutations in this gene can have significant consequences for cellular function. The SPOP gene consists of multiple exons and introns. Exons (Table 1) are the coding regions of the gene that are transcribed into messenger RNA (mRNA) and eventually translated into the SPOP protein. Introns are noncoding regions that are removed during mRNA processing. The gene structure includes several exons that are transcribed into mRNA and subsequently translated into the SPOP protein. The precise organization of exons and introns may vary among species, but in humans, the SPOP gene generally consists of eight exons. Here is a brief overview of the exons in the SPOP gene:

The SPOP protein (Figure 2) plays a critical role in protein degradation pathways, particularly by acting as a substrate adaptor for the Cullin3-RING E3 ligase complex. This complex target various downstream signaling pathways involved in cellular proliferation and survival. SPOP's substrate-binding cleft, encoded by exons 2 and 3, is essential for its recognition of target proteins. SPOP has a modular structure that plays a pivotal role in cellular functions, particularly in protein homeostasis and degradation [3].

The domains of the SPOP include:

a. N-terminal MATH domain: The MATH (Meprin and TRAF homology) domain is located within the central region of the SPOP protein. This domain is responsible for substrate recognition and binding. It plays a pivotal role in mediating the interaction between SPOP and its target proteins, which are subsequently targeted for ubiquitination and degradation.
b. BTB domain: The BTB (Bric-a-brac, Tramtrack, Broad-complex) domain is located at the N-terminal region of the SPOP protein. It functions as a protein–protein interaction domain, allowing SPOP to interact with other proteins and form complexes. This domain is involved in the assembly of the Cullin3-RING E3 ligase complex, which plays a crucial role in protein degradation pathways.
c. BACK domain: The BACK (BTB and C-terminal Kelch) domain is situated between the MATH domain and the POZ domain within the SPOP protein. This domain contains a series of Kelch-like repeats that are involved in protein–protein interactions and substrate recognition. The BACK domain is known to interact with specific protein targets, contributing to the substrate specificity of SPOP. It plays a crucial role in facilitating the assembly of the Cullin3-based E3 ubiquitin ligase complex, which is responsible for targeting proteins for ubiquitin-mediated degradation.
d. C-terminal nuclear localization sequence (NLS): The C-terminal region of the SPOP protein contains a NLS. This sequence is responsible for directing the protein to the cell nucleus. The NLS allows SPOP to enter the nucleus, where it can participate in various cellular processes, including gene regulation and protein degradation. The nuclear localization of SPOP is essential for its interactions with other nuclear proteins and for executing its functions related to substrate recognition and protein degradation.

The SPOP protein's structural domains collectively contribute to its role as a substrate adaptor in the ubiquitin-proteasome system. In the context of prostate cancer, mutations within the substrate-binding cleft of the SPOP protein have been linked to aberrant protein interactions and degradation pathways, potentially contributing to cancer development. SPOP mutations predominantly affect the MATH domain of the protein (Table 2) [14].

### 4.2. Prevalence of SPOP Mutations in Prostate Cancer

The prevalence of these mutations varies across different prostate cancer subtypes, suggesting potential implications for targeted therapies. The overall frequency of SPOP mutations in prostate cancer is approximately 8.1% (ranging from 4.6% to 14.4%) based on a study by the authors of [18–20]. These mutations were found in a subset of prostate cancer cases. Extensive genomic studies and clinical data revealed that these mutations occur in a significant subset of prostate cancer cases, particularly in certain molecular subtypes characterized by genomic instability and chromosomal rearrangements. SPOP mutations are identified as early events in the development of a specific subtype of prostate cancer. This suggests that these mutations occur relatively early in the progression of the disease and may play a fundamental role in its initiation. The presence of SPOP mutations is linked to a notably high frequency of genomic rearrangements within prostate cancer cells. Genomic rearrangements can lead to significant alterations in the DNA structure, potentially contributing to cancer development and progression. When SPOP mutations occur, it leads to the loss of the MAP3K7 and CHD1 proteins. These proteins are involved in various cellular processes, and their loss may lead to tumor development and progression. Both MAP3K7 and CHD1 proteins are independently associated with high rearrangement frequencies. This implies that the loss of each of these proteins can independently contribute to genomic instability, one of the cancer hallmarks. The clonality model suggests a temporal sequence of events in the context of SPOP mutations, MAP3K7 loss, and CHD1 loss. According to this model, SPOP mutations precede MAP3K7 loss, which in turn precedes CHD1 loss. This sequence of events sheds light on the chronological order of molecular alterations in this subtype of prostate cancer [21, 22]. MAP3K7 loss occurs at a relatively high frequency of around 30%, followed by CHD1 loss at approximately 15%. In contrast, SPOP mutations occur at a lower frequency, approximately 10% [23].

Cavalcante et al. [24] found that SPOP mutations exhibit distinct patterns and implications in different cancer types, indicating that SPOP mutations drive distinct molecular and immune landscapes in prostate and endometrial cancers [24]. In prostate cancer, they appear to result in a loss-of-function mechanism, acting as Tumour suppressor, while in endometrial cancer, they may represent a gain-of-function mechanism. These insights suggest the potential for tailored therapeutic approaches based on SPOP mutation status in these cancers. In prostate cancer, these mutations were identified in approximately 9.2% of cases, whereas in endometrial cancer, they were found in approximately 4.3% of cases [24, 25]. SPOP mutations also led to alterations in epigenetic gene expression, including the opposite regulation of BRD2 transcripts [24]. It modulates transcription of genes involved in cell cycle progression and apoptosis. In prostate cancer, SPOP mutations disrupt the ubiquitination process, leading to aberrant stabilization and altered expression of epigenetic regulators, including BRD2 [24]. Consequently, dysregulated BRD2 may enhance oncogene transcription and promote tumor progression, contributing to therapy resistance.

### 4.3. Molecular Alterations in SPOP-Mutant Prostate Cancers and Disease Characterization

SPOP mutations display distinct signature patterns, often involving hotspot mutations at residues F133 and F102. These mutations are associated with altered protein–protein interactions and changes in substrate specificity, impacting the ubiquitination pathway [26]. SPOP mutations disrupt the normal function of the E3 ubiquitin ligase substrate adaptor protein, leading to impaired substrate recognition. The well-documented consequence is the dysregulation of the AR signaling pathway. Mutant SPOP fails to effectively ubiquitinate AR, resulting in increased AR protein stability and enhanced transcriptional activity, contributing to prostate cancer progression. Tumors characterized by SPOP mutations exhibit distinctive molecular features. Notably, they are often mutually exclusive with ETS gene fusions, although some cases may possess co-occurring FOXA1 mutations. This unique molecular profile contributes to the heterogeneity of SPOP-mutant prostate cancers. The complex process of cancer progression is influenced by numerous genetic and molecular abnormalities; people with tumors have been shown to have more genetic mutations [27].

SPOP mutations are predominantly clustered in the MATH domain, with specific hotspot residues such as F133 and F102 frequently mutated in prostate cancer [28]. These hotspots are critical as they influence SPOP's ability to bind substrates and regulate downstream pathways, including AR signaling and chromatin remodeling. The clustering of mutations in these regions underscores their functional importance in tumorigenesis, as evidenced by their association with genomic instability and altered epigenetic regulation. Interestingly, certain regions of the SPOP gene remain mutation-free (NIL), even in large-scale genomic studies. This absence of mutations in specific regions suggests that these areas may not play a significant role in tumorigenesis or could be functionally redundant. Alternatively, these regions might be structurally essential for maintaining protein integrity, making them intolerant to mutations. This phenomenon highlights the selective pressure on specific domains of SPOP during cancer progression, emphasizing the importance of hotspot mutations while leaving other regions unaltered.

Prostate cancer is marked by a diverse genetic landscape, encompassing both germline and somatic mutations [29]. Germline mutations, such as those in BRCA1, BRCA2, and HOXB13 genes, can confer an increased risk of prostate cancer. Somatic mutations in genes like TP53, PTEN, and SPOP are frequent drivers of disease progression [30]. Within the realm of primary prostate cancers, there is a notable occurrence of recurring mutations in genes such as TP53, SPOP, and FOXA1 [31]. Additionally, it is common to observe deletions affecting RB1, PTEN, and CHD1, as well as amplifications involving MYC [32]. Furthermore, ETS family gene fusions, including the well-known TMPRSS2-ERG fusion, are prevalent. The utilization of comprehensive genomic sequencing has provided valuable insights into the spectrum of alterations in metastatic castration-resistant prostate cancer (mCRPC), shedding light on the genomic landscape. Interestingly, even within this subgroup of metastatic tumors, the mutation rate remains relatively low, hovering at three to four mutations per megabase (Mb) [6, 33]. Somatic mutations in mCRPC tumors manifest in several genes that experience relatively rare alterations in primary prostate cancers. These genes encompass PIK3CA, PIK3CB, RSPO, BRAF, RAF1, APC, CTNNB1, and ZBTB16 (PLZF) [34].

SPOP has also been identified as an enzyme that plays a crucial role in enabling homology-directed DNA repair (HDR) of double-strand breaks (DSBs) in the DNA [35]. HDR is a precise and error-free repair pathway that relies on the availability of homologous DNA sequences as templates to repair damaged DNA. Mutations in the SPOP gene can disrupt its normal function as an HDR regulator [36]. When SPOP is mutated, it fails to promote HDR effectively, which means that the cell's ability to use the accurate repair pathway is compromised. In the absence of functional HDR, cells tend to favor the nonhomologous end joining (NHEJ) pathway for DNA repair. NHEJ is a more error-prone mechanism compared to HDR and can result in the introduction of small insertions or deletions at the site of DNA repair. The shift towards NHEJ as the favored repair pathway in SPOP-mutant cells leads to a high degree of intrachromosomal breaks and erroneous DNA repair. This increased genomic instability can result in a higher frequency of genomic rearrangements, including deletions, duplications, and translocations, contributing to the genomic instability associated with SPOP mutations. Overall, the failure of SPOP to promote HDR and the preference for NHEJ in SPOP-mutant cells have significant implications for DNA repair fidelity and genomic stability. This mechanism sheds light on how SPOP mutations can lead to genomic rearrangements, which are a hallmark of certain cancer subtypes, including prostate cancer [35, 36].

Alterations in the AR are prevalent in mCRPC but rarely appear in primary, treatment-naïve prostate cancer [37]. These AR alterations are closely linked to resistance against androgen therapy. They encompass single-nucleotide variations (SNVs), amplification of the AR gene locus, structural rearrangements of AR, and amplifications affecting an AR enhancer. Deletions and mutations involving PTEN and TP53 are present in a substantial proportion, ranging from 40% to 50%, of mCRPC cases [38]. A noteworthy fraction of patients also carries deficiencies in DNA repair proteins, potentially making them responsive to poly(ADP-ribose) polymerase (PARP) inhibitor therapy. Moreover, patients with metastatic prostate cancer exhibit a notable occurrence of germline DNA repair alterations, with a frequency as high as 12%. These findings hold potential implications not only for cancer therapy but also for prostate cancer prevention. Additionally, ETS family gene fusions are a characteristic feature, being present in approximately 50% of both primary and metastatic prostate cancers. Genomic rearrangements involving ETS family genes, predominantly TMPRSS2-ERG fusion, are recurring events in prostate cancer, further shaping its genetic identity. Epigenetic modifications, encompassing DNA methylation and histone modifications, exert a profound influence on prostate cancer progression. These modifications can silence tumor suppressor genes and activate oncogenes, thus contributing to cellular transformation and therapy resistance. Emerging evidence suggests that prostate cancer can be classified into distinct molecular subtypes based on their genomic and molecular characteristics [39]. These subtypes, such as luminal, basal, and neuroendocrine, have varying responses to therapies and clinical outcomes, highlighting the importance of tailored treatment strategies. Advancements in genomic technologies have led to the identification of promising biomarkers for prostate cancer diagnosis, prognosis, and treatment prediction. Liquid biopsies, utilizing circulating tumor DNA, RNA, and proteins, hold potential for noninvasive monitoring of disease progression and therapeutic response.

The AR pathway plays a pivotal role, regulating prostate growth and differentiation. Dysregulation of AR signaling, often seen in advanced prostate cancer, contributes to treatment resistance and disease progression [9]. The biochemical activity of androgen is started by testosterone or DHT attaching to its specific receptor, the AR, activating it. When unattached from its ligand, AR is found in the cytoplasm as a component of an inactive complex that also contains the heat shock proteins HSP40, HSP70, and HSP90 [40] and other chaperone proteins to protect the receptor against degradation. These proteins play a crucial function in maintaining an accessible AR shape and preventing premature AR degradation. The AR undergoes a conformational change when it binds to a particular ligand, creating a more compact and stable form of the receptor. When AR is activated, it separates from heat shock proteins and moves to the nucleus, engaging in homodimer interactions with DNA androgen response elements [41]. A second dimerization interface is needed to bind to an AR response element since AR employs the DBD to bind to ARE elements. The protein–protein and protein–DNA connections that allow for this unexpected structure are preserved, and some AR-specific dimerization contacts are responsible for the AR specificity of AR response elements. In order to control the gene-specific expression that affects the development and survival of target cells, the active DNA-bound AR can entice co-regulator proteins and fundamental transcriptional machinery [42]. In addition to aiding PCa cells in promoting development and continued existence, AR signaling can also contribute to the development of resistance to ADT. This occurs through a variety of mechanisms, including AR gene mutations or amplifications, increased AR expression, and activation of alternative signaling pathways. As a result, targeting the AR signaling pathway is an important therapeutic strategy in the therapy of PCa, and there are several drugs that specifically target AR signaling, such as enzalutamide, abiraterone, and apalutamide.

Additionally, research has demonstrated that AR interacts with other signaling pathways, such as the PI3K/Akt pathway [43]. Phosphatidylinositol 3-kinase (PI3K) and AR signaling are two crucial pathways that control cellular growth, survival, and metabolism. Crosstalk between these two pathways has been connected to the development and progression of numerous cancers, including PCa. Cell growth, metabolism, and survival require the stimulation of the PI3K signaling pathway, which is triggered by a number of growth hormones and cytokines. Phosphatidylinositol-3,4,5-trisphosphate (PIP3) is produced as a result of PI3K activation, and PIP3 then recruits and activates a number of downstream effectors, such as Akt and mTOR. Akt promotes cell survival and growth by inhibiting apoptotic pathways and activating signaling pathways that promote protein synthesis and glucose metabolism. mTOR, on the other hand, promotes cell growth and proliferation by regulating protein synthesis and autophagy. Contrarily, androgen binding to the receptor activates AR signaling, which then causes the transcriptional induction of gene products necessary for survival and development of cells. Previous research has linked SHH signaling to prostate cancer progression and resistance to hormone therapy [15]. However, little was known about the specific role of GLI3, a downstream effector in the SHH pathway, in this process. GLI3 is found to be expressed at significantly higher levels in prostate cancer cell lines, tumor xenografts, and human prostate tumor tissues [15]. GLI3 mRNA and protein levels are negatively regulated by androgens. When androgens are withdrawn, GLI3 levels increase in prostate cancer cells, contributing to the development of androgen-independent growth. Targeted depletion of GLI3 in prostate cancer cells prevents the acquisition of androgen-independent growth. It also impairs cell growth and migration, even after androgen independence is established. Additionally, GLI3 knockdown blocks the formation of castration-resistant tumors and their metastatic spread in mice. E3 ubiquitin ligase adaptor protein SPOP targets GLI3 for ubiquitin-mediated proteasomal degradation in prostate cancer cells. Mutations in the SPOP gene, which are frequently found in primary prostate cancer, stabilize GLI3, leading to its pathologic accumulation [15]. GLI3 and AR appear to cooperate in promoting androgen-independent growth in SPOP-mutant prostate cancer cells. Depletion of either GLI3 or AR inhibits androgen-independent growth in these cells, indicating a possible coordinated transcriptional control. Some key implications of this molecular events for disease characterization are:

a. Mutation frequency: SPOP mutations are frequent in prostate cancer, with prevalence ranging from 10% to 15% across different ethnic and demographic backgrounds [17]. These mutations are often early events in the development of prostate cancer and contribute to its molecular heterogeneity.
b. Genomic rearrangements: Mutant SPOP has been associated with a high degree of genomic instability and rearrangements in prostate cancer. SPOP mutations can lead to defects in HDR, favoring the more error-prone NHEJ pathway, resulting in genomic rearrangements [17].
c. Activation of signaling pathways: SPOP mutations activate various signaling pathways in prostate cancer. Notably, they can activate the PI3K/mTOR pathway, promoting cell proliferation and survival. Moreover, SPOP mutations lead to an activation of the AR signaling pathway, which is central to prostate cancer progression. Mutant SPOP stabilizes AR, contributing to disease development and progression.
d. Interaction with other genomic alterations: SPOP-mutated prostate cancers often exhibit distinct genomic alterations. For instance, they are frequently mutually exclusive with ETS fusions but may co-occur with mutations in other genes such as FOXA1. These additional genomic alterations can contribute to the molecular and clinical heterogeneity of SPOP-mutated prostate cancer [30].

Early detection remains vital for improving prostate cancer outcomes. Screening methods such as the prostate-specific antigen (PSA) test and digital rectal examination aid in identifying potential cases. However, debates continue regarding the balance between overdiagnosis and the benefits of early detection. The identification of novel biomarkers associated with prostate cancer has the potential to transform diagnosis and treatment. Biomarkers linked to AR signaling and genetic mutations offer insights into disease aggressiveness and treatment response [44]. Targeted therapies, including AR pathway inhibitors, show promise in managing advanced and CRPC. The intricate interplay of genetic and molecular alterations in prostate cancer underscores the importance of precision medicine approaches. Prostate cancer represents a multifaceted challenge necessitating comprehensive research, improved screening strategies, personalized treatment approaches, and enhanced survivorship care. A holistic approach that integrates scientific advancements with patient-centered care is essential for combating this complex disease and improving patient outcomes. The genetic and molecular landscape of prostate cancer is complex and multifaceted. Unraveling these intricacies holds the promise of enhancing our understanding of disease mechanisms, refining diagnostic and prognostic approaches, and guiding the development of targeted therapies tailored to the unique molecular profiles of patients.

### 4.4. Computational Strategies for Targeted Interventions Leveraging TCGA cBioPortal Database

**Figure 3:**
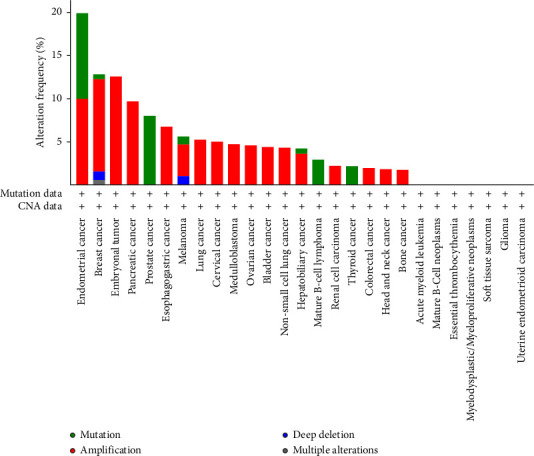
SPOP mutations across different cancer types from the cBioPortal database (https://www.cbioportal.org/).

A Pan-cancer analysis of whole genomes data from the cBioPortal database (https://www.cbioportal.org/) has revealed the presence of SPOP modifications in various types of tumors [45]. These modifications encompass a range of changes such as mutations, structural variations, amplifications, deep deletions, and multiple alterations, as illustrated in Figure 3. Interestingly, amongst the various cancers illustrated in Figure 2, only three cancers, namely prostate cancer, mature B-cell lymphoma, and thyroid cancer, had mutations as the only alteration, with prostate cancer being the highest alteration frequency amongst the three cancers.

**Figure 4:**
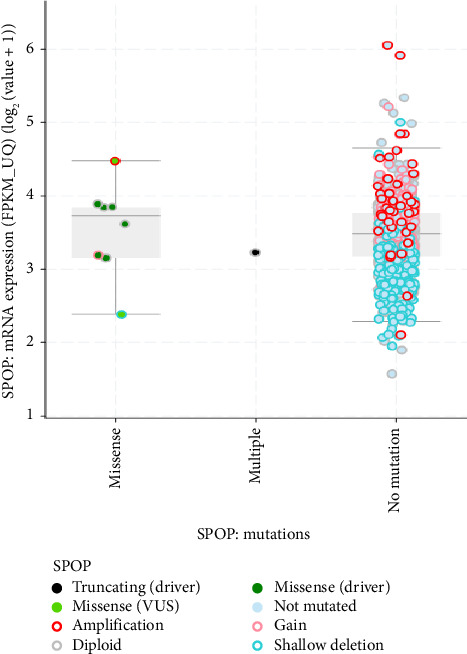
Type of mutation based on mRNA expression from the cBioPortal database (https://www.cbioportal.org/).

The mRNA expression data also provides valuable insights into the mutation types and effects of mutations in the SPOP gene (Figure 4) [45]. Mutations in the SPOP gene can disrupt its normal function as a ubiquitin ligase, leading to alterations in mRNA expression levels. By analyzing mRNA expression profiles, researchers can identify genes and pathways that are either upregulated or downregulated due to SPOP mutations. This differential expression can indicate the impact of specific mutation types. Different mutation types within the SPOP gene may result in distinct signature mRNA expression patterns. For example, certain mutations may lead to the dysregulation of genes involved in AR signaling in prostate cancer. These signature expression patterns can serve as biomarkers for identifying specific mutation types. This mRNA expression data can provide insights into the functional consequences of SPOP mutations. Mutations that disrupt SPOP's ability to degrade target proteins via ubiquitination may lead to the accumulation of specific proteins. mRNA expression data can reveal changes in the expression of these target genes, indicating the functional impact of SPOP mutations. By examining mRNA expression data in conjunction with SPOP mutation status, researchers can classify tumors into different subtypes based on their gene expression profiles. This classification may help in understanding the heterogeneity of SPOP-mutated cancers and tailoring treatment strategies accordingly [46].

### 4.5. Types and Distribution of SPOP Mutations

Mutations in the SPOP gene have been identified in various cancers, including prostate cancer. These mutations can result in altered SPOP protein function and have implications for disease development. Here are some of the mutations found in the SPOP gene:

a. Missense mutations: Missense mutations involve the substitution of one nucleotide for another, leading to the replacement of a single amino acid in the SPOP protein. These mutations can affect the protein's structure and function.
b. Truncating mutations: Truncating mutations, including nonsense mutations and frame-shift mutations, result in the premature termination of the SPOP protein. These mutations often lead to nonfunctional or partially functional SPOP.
c. Hotspot mutations: Hotspot mutations are recurrent mutations that occur more frequently in specific regions of the SPOP gene. These mutations are of particular interest because of their higher prevalence and potential impact on SPOP's regulatory roles.
d. Single nucleotide polymorphisms (SNPs): While not necessarily pathogenic, SNPs in the SPOP gene can be associated with a person's susceptibility to certain diseases, including prostate cancer.
e. Copy number alterations: In addition to point mutations, alterations in the copy number of the SPOP gene have been observed in some cancer cases. These alterations can lead to changes in SPOP expression levels.
f. Structural variants: Structural variants, such as gene fusions involving the SPOP gene, have been reported in prostate cancer. These rearrangements can result in the fusion of SPOP with other genes, potentially leading to altered protein function.

**Figure 5:**
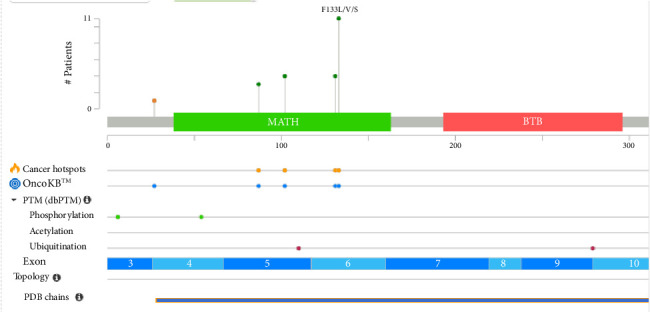
Combine study of SPOP mutation gene from the cBioPortal database (https://www.cbioportal.org/).

In the extended analysis conducted using the cBioPortal database (Figure 5), a comprehensive examination of combined data from three separate studies, involving a total of 615 samples obtained from 440 patients, was performed. We observed in this dataset that 24 out of the 440 queried patients exhibited SPOP mutations corresponding to a mutation prevalence of approximately 5% among the studied patient population. Additionally, when considering the individual samples, 34 out of the 615 queried samples showed SPOP mutations, resulting in a mutation prevalence of approximately 6%. It is noteworthy that among the identified mutations, there were 10 cases of duplicate mutations observed in patients who had multiple samples analyzed. This suggests that these specific mutations were recurrent within the same patients, possibly indicating a consistent genetic alteration pattern. Moreover, the identification of duplicate mutations within certain patients underscores the significance of specific SPOP mutation events, which may have implications for understanding the genetic landscape of the studied cohort and its potential relevance to disease mechanisms or treatment responses.

It is important to note that the specific mutations in the SPOP gene can vary among different cancer types and individual patients. The functional consequences of these mutations are an active area of research, as they can impact key cellular processes and pathways, including those related to cancer development and progression.

### 4.6. Clinical and Therapeutic Implications of SPOP Mutations

SPOP mutations are integral to the characterization of prostate cancer and the development of targeted interventions. They contribute to genomic instability, activate signaling pathways, and interact with other genomic alterations [47]. Understanding the role of SPOP in regulating the AR signaling pathway is critical for tailoring treatments, particularly ADT, and exploring therapeutic strategies targeting SPOP and its downstream effects in AR signaling. This research is advancing our understanding of prostate cancer and offering new avenues for precision medicine in its management. In the context of prostate cancer, SPOP targets the AR for ubiquitylation and degradation. This regulation of AR stability leads to reduced AR activity, which is a key driver of prostate cancer [48]. SPOP mutations can impact the response to ADT, a common treatment for advanced prostate cancer. Understanding a patient's SPOP mutation status can help guide treatment decisions. Some studies suggest that SPOP-mutated prostate cancers may have distinct responses to ADT, which could influence clinical outcomes [49].

The prognostic significance of SPOP mutations in prostate cancer remains an area of active research. Some studies suggest that SPOP mutations may be associated with favorable clinical outcomes, while others report associations with adverse outcomes [47]. The context, co-occurring genetic alterations, and disease stage may influence these variable clinical effects. SPOP mutations have opened new avenues for targeted therapy development. Efforts to exploit the dependence of SPOP-mutant prostate cancer cells on dysregulated AR signaling are ongoing, with small molecule inhibitors and gene-editing techniques being explored as potential therapeutic strategies. By analyzing ctDNA from blood or urine samples, researchers aim to identify specific genetic alterations associated with PCa [50]. These alterations can serve as indicators of the presence and progression of the disease [50]. Chen et al. [50] recognized the value of combining multiple ctDNA markers, emphasizing that this approach may improve the accuracy of PCa diagnosis. By examining a panel of genetic changes, researchers can achieve higher sensitivity and specificity, reducing the likelihood of false-positive or false-negative results. This endeavor holds the potential to offer substantial information for the development of personalized treatment strategies tailored to patients exhibiting specific SPOP mutation subtypes.

The identification of GLI3 as a key player in the development and progression of CRPC opens up new avenues for therapeutic interventions. The link between SPOP mutations and the stabilization of GLI3 provides insights into the genetic basis of prostate cancer. GLI3 levels are significantly higher in prostate tumors with SPOP mutations. The study suggests that factors regulating GLI3 expression and activity could significantly impact the development and progression of prostate cancer, particularly in the context of SPOP mutations. Targeting GLI3 or the SHH pathway could be explored as potential strategies to combat CRPC. Understanding the role of GLI3 in promoting androgen-independent growth is crucial. Many prostate cancer treatments focus on suppressing androgen signaling, but CRPC often becomes resistant to these therapies. Targeting GLI3 may help address this resistance and develop more effective treatments for advanced prostate cancer. This knowledge may lead to the development of personalized treatments or therapies targeting specific genetic alterations in prostate cancer patients. Recent research has confirmed intriguing insights into the role of SPOP mutations in mCRPC and their implications for disease management [46]. Notably, patients with mCRPC bearing SPOP mutations often exhibit a concurrent deletion of CHD1, rendering them highly sensitive to abiraterone treatment, a significant advancement in targeted therapy [46]. Moreover, the level of SPOP expression emerges as a critical determinant of prognosis, with lower expression levels associated with poorer outcomes. SPOP mutations exert their influence on disease progression and prognosis by modulating androgen signaling. This multifaceted impact includes alterations in vital processes such as steroid hormone biosynthesis, fatty acid metabolism, adipogenesis, and androgen response, and cholesterol homeostasis—crucial facets of androgen metabolism. These alterations can disrupt not only androgen synthesis but also the biology of androgens and AR function.

In the context of radiation therapy, current evidence suggests that patients with SPOP mutations may exhibit increased basal reactive oxygen species (ROS) levels [51]. This phenomenon could reduce the sensitivity to radiation therapy, possibly through mechanisms such as PTEN inhibition, PI3K/AKT pathway activation, and ROS reduction. As a result, alternative treatment modalities may be more appropriate for this patient subgroup. However, it is important to note that SPOP mutation status does not necessarily correlate with the expression of SPOP mRNA in prostate cancer tissue. Survival analysis indicates that SPOP mutations alone do not predict better or worse prognosis [14, 46]. Instead, the mRNA expression level of SPOP emerges as a critical factor, with lower levels associated with significantly worse disease prognosis. This highlights the potential for tailoring treatment strategies in prostate cancer based on SPOP mutation status.

## 5. Conclusions

This review highlights the significance of SPOP mutations in prostate cancer, emphasizing their role in disrupting key cellular pathways and influencing clinical outcomes. SPOP mutations are characterized by hotspot residues and distinct genomic profiles, which have implications for therapeutic strategies. Notably, patients with SPOP mutations often exhibit favorable responses to ADT, although concurrent mutations can affect prognosis. These findings underscore the potential of SPOP mutations as biomarkers for personalized treatment approaches in prostate cancer management.

## Figures and Tables

**Table 1:** Coding exons of SPOP gene.

Exon	Function
1	The first exon is noncoding and often contains regulatory elements that control gene expression and transcription.
2	This exon encodes the N-terminal region of the SPOP protein, including a substrate-binding cleft and a BTB domain, which is involved in protein–protein interactions.
3	Encodes additional portions of the N-terminal region of the SPOP protein.
4	Contains sequences coding for the middle region of the SPOP protein.
5	Encodes the central portion of the protein, including the MATH domain, which is involved in substrate binding.
6	Encodes more of the central region of the SPOP protein.
7	Contains sequences coding for the C-terminal region of the SPOP protein.
8	The last exon encodes the final part of the C-terminal region of the SPOP protein.

**Table 2:** SPOP gene mutations.

SPOP domain	Nucleotide substitution	Amino acid substitution	Reference
MATH^∗^	T > G	Y87C	[13, 14]
	C > T	F102C	[14, 15]
	C > T	F102L	[13]
	G > A	F133L	[16]
	C > G	F133V	[14, 15]
	G > C	F125C	[16]
	T > A	W131R	[16]
	T > G	W131G	[14]

BTB	C > G	Y87C	[17]

BACK	NIL	NIL	NIL

NLS	NIL	NIL	NIL

*Note:* NIL (No discovered mutation).

^∗^Found to be hotspot for various mutations.

## Data Availability

Data sharing is not applicable to this article as no datasets were generated or analyzed during the current study.
